# p21-Dependent Senescence Induction by BMP4 Renders Glioblastoma Cells Vulnerable to Senolytics

**DOI:** 10.3390/ijms26093974

**Published:** 2025-04-23

**Authors:** Mia Niklasson, Erika Dalmo, Anna Segerman, Veronica Rendo, Bengt Westermark

**Affiliations:** 1 Department of Immunology, Genetics and Pathology, Rudbeck Laboratory, Uppsala University, 751 85 Uppsala, Sweden; erika.dalmo@igp.uu.se (E.D.); anna.segerman@igp.uu.se (A.S.); veronica.rendo@igp.uu.se (V.R.); bengt.westermark@igp.uu.se (B.W.); 2 Department of Medical Sciences, Cancer Pharmacology and Computational Medicine, Uppsala University Hospital, 751 85 Uppsala, Sweden

**Keywords:** glioblastoma, senescence, BMP4, mesenchymal transition, p21

## Abstract

Glioblastoma (GBM) is a highly malignant brain tumor with extensive cellular heterogeneity and plasticity. Bone morphogenetic protein 4 (BMP4) has shown potential as a therapeutic agent by promoting differentiation, but its effects are complex and context dependent. While BMP4’s role in differentiation is well established, its impact on senescence remains unclear. This study investigates BMP4’s ability to induce senescence in GBM cells. Primary GBM cultures were treated with BMP4 and analyzed for senescence markers, including cell enlargement, p21 expression, senescence-related gene enrichment, and senescence-associated-β-galactosidase activity. A p21 knockout model was used to determine its role in BMP4-induced senescence, and sensitivity to the senolytic agent navitoclax was evaluated. BMP4 induced senescence in the GBM cultures, particularly in mesenchymal (MES)-like GBM cells with high baseline p21 levels. The knockout of p21 nearly abolished BMP4-induced senescence, maintaining cell size and proliferation. Furthermore, navitoclax effectively eliminated BMP4-induced senescent cells through apoptosis, while sparing cells with normal p21 expression. Our findings highlight BMP4 as an inducer of p21-dependent senescence in GBM, particularly in MES-like cells. This study clarifies BMP4’s dual roles in differentiation and senescence, emphasizing their context dependence. Given the strong link between MES-like cells and therapy resistance, their heightened susceptibility to senescence may aid in developing targeted therapies for GBM and potentially other cancers with similar cellular dynamics.

## 1. Introduction

Glioblastoma (GBM) is one of the most aggressive cancers, characterized by rapid and infiltrative growth, treatment resistance, and significant intratumoral heterogeneity. A major challenge in GBM therapy is the plasticity of malignant cells, which allows them to shift between distinct cellular states [1,2,3], particularly along a spectrum from neurodevelopmental/proneural (PN) to injury-response/mesenchymal (MES) molecular subtypes [4,5]. This plasticity is closely associated with therapy resistance, as recurrent GBM tumors frequently exhibit a MES-like phenotype, which correlates with poorer clinical outcomes [6,7].

Bone morphogenetic protein 4 (BMP4), a growth factor with anti-proliferative properties, has been proposed as a therapeutic option for GBM since it downregulates stemness markers like SOX2, inhibits tumor growth, and induces (reversible) astrocytic differentiation [8,9,10,11]. A phase I clinical trial using BMP4 for recurrent GBM reported partial or complete responses in 20% of patients [12], highlighting its clinical promise. However, these encouraging outcomes also underscore the variability in BMP4 responsiveness, previously observed among patient-derived GBM cell cultures [8,13]. Moreover, emerging evidence suggests that BMP4 may also have adverse effects, including promoting MES transition [3] and increasing resistance to standard-of-care treatment [14].

BMP4 has also been shown to induce cellular senescence—a state of permanent cell-cycle arrest—in lung cancer cells [15], but its potential to trigger senescence in GBM remains underexplored. This represents a critical knowledge gap, particularly given the dual role senescence can play in cancer progression (reviewed in [16]). Senescence can be induced by various factors, including telomere shortening, oncogene activation, radiation, oxidative stress, chemotherapy, cell enlargement, and cytokine signaling [17,18,19,20,21,22,23,24]. While senescence can serve as a tumor-suppressive barrier by halting the proliferation of damaged cells, it also contributes to a pro-tumorigenic environment through the senescence-associated secretory phenotype (SASP)—a collection of inflammatory cytokines, proteases, and extracellular matrix components (reviewed in [25]). These SASP factors can promote tumor growth and recurrence. Similarly, therapy-resistant MES-like GBM cells secrete SASP-like factors [4], linking therapy resistance to pro-inflammatory signaling. Whether and how BMP4 influences the interplay between MES-like and senescent GBM cell states remains unclear.

In this study, we explore the ability of BMP4 to induce senescence in GBM cells and investigate its potential link to MES characteristics. We examine the differential responses of PN- and MES-like cells from the same tumor to BMP4, with a focus on the role of the cyclin-dependent kinase (CDK) inhibitor p21^Waf1/Cip1^ (p21) in this process. Our findings reveal that MES-like cells, characterized by high basal levels of p21, are more susceptible to BMP4-induced senescence, which relies on p21 activation. Furthermore, we show that senolytic treatments can selectively eliminate these senescent cells by inducing apoptosis. This study provides new insights into BMP4’s dual roles in differentiation and senescence induction, underscoring the importance of understanding these mechanisms in a context-dependent manner when evaluating BMP4 as a therapeutic option for glioblastoma.

## 2. Results

### 2.1. BMP4 Induces a Senescence-like Phenotype in GBM Cells

**Figure 1:**
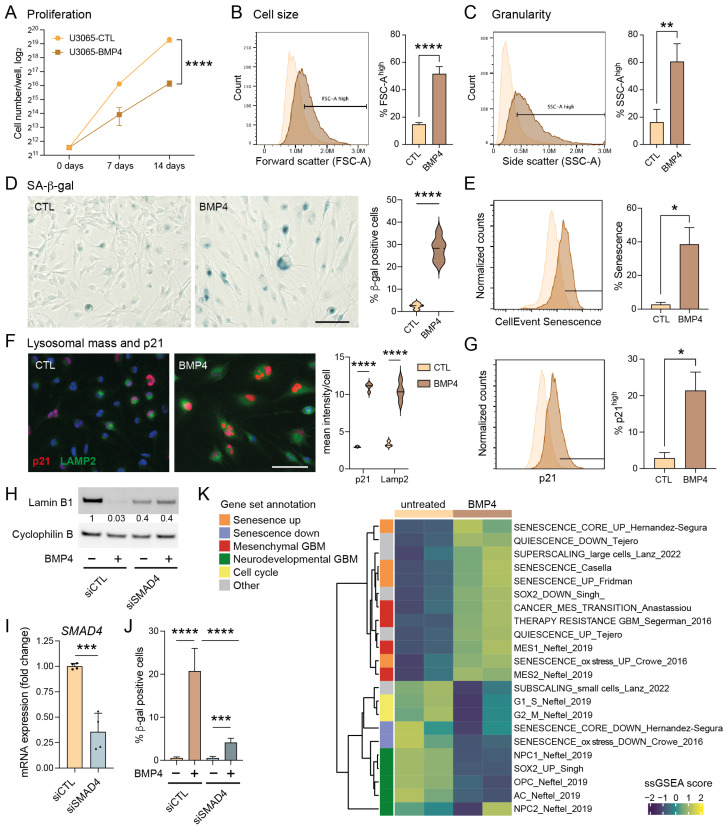
The BMP4-SMAD signaling pathway induces cell-size enlargement and a senescence-like phenotype in GBM cell cultures. (**A**) Proliferation curves of U3065MG cells (cells counted on days 0, 7 and 14), untreated (CTL) or treated with BMP4 (10 ng/mL). Unpaired *t*-test, ****, *p* < 0.0001. (**B**–**J**) U3065MG cells treated +/−BMP4 for two weeks. (**B**,**C**) Histograms (left) showing cell-size measurement (**B**) and granularity (**C**) using flow cytometry forward scatter (FSC-A) and side scatter (SSC-A) area values, respectively, and the quantification (right) of the FSC-A and SSC-A high populations of quadruplicate experiments. Unpaired *t*-test, ****, *p* < 0.0001; **, *p* = 0.0015. (**D**) Left, photographs of U3065MG cells +/−BMP4 stained for SA-β-gal—scale bar 100 µm; right, the quantification of SA-β-gal-positive cells. ****, *p* < 0.0001. (**E**) Histograms of cells stained for SA-β-gal (CellEventSenescence kit, Cell Signaling Technology, Danvers, MA, USA) and analyzed by flow cytometry, left; and the quantification of the CellEventSenescence high cell population (gate in histogram) from two experiments, right. *, *p* ≤ 0.05. (**F**) p21 and lysosomal marker LAMP2 immunofluorescence staining (left) and quantification (right). ****, *p* < 0.0001. (**G**) Histograms of cells stained with p21 antibody and analyzed by flow cytometry, left; and the quantification of cells with high p21 expression (two experiments), right. *, *p* ≤ 0.05. (**H**–**J**) *SMAD4* knockdown experiment in U3065MG using siRNA for 14 days +/−BMP4 (12 days). siCTL, non-targeting control siRNA. (**H**) Lamin B1 protein levels using Western blot analysis. The quantification of protein-band intensities is shown, with values normalized to loading control cyclophilin B. (**I**) *SMAD4* mRNA expression using qRT-PCR analysis. Two experiments, unpaired *t*-test, ***, *p* = 0.0004. (**J**) The quantification of SA-β-gal positive cells in *SMAD4* knockdown experiment. See Supplementary Figure S1D for photographs. Unpaired *t*-test, ***, *p* < 0.001; ****, *p* < 0.0001. (**K**) A heatmap of single-sample GSEA (ssGSEA) using gene sets related to senescence, quiescence, GBM subtypes, GBM multitherapy resistance, cell size, and cell cycling on transcriptome data from untreated or BMP4-treated (14 days) U3065MG cells—two experiments.

To investigate BMP4’s potential to induce senescence in GBM, we utilized the primary human GBM cell line U3065MG, a highly heterogeneous and plastic cell model previously characterized by single-cell RNA sequencing of barcoded cells and clonal cultures [3,6]. Treatment with recombinant human BMP4 (10 ng/mL, fresh addition every third day) for up to two weeks significantly reduced cell proliferation rate (Figure 1A), consistent with our previous findings [8]. Bulk RNA sequencing and Gene Set Enrichment Analysis (GSEA) revealed that BMP4-treated cells showed a significant enrichment of gene sets related to cellular senescence and the mesenchymal (MES) GBM subtype—ranking among the top 20 of 2389 gene sets from the MSigDB chemical and genetic perturbations collection—alongside a reduction in proliferation-related transcripts and proneural (PN) GBM markers (Supplementary Table S1).

To validate the induction of senescence by BMP4, we conducted a series of senescence-related assays [26,27,28]. Unlike quiescent cells, senescent cells typically exhibit abnormal cellular growth, i.e., cell-size enlargement, and an increase in granularity, both of which we observed following BMP4 treatment (Figure 1B,C, respectively). Furthermore, staining for senescence-associated (SA)-β-gal revealed an induction in approximately 20–40% of the cells (Figure 1D,E). Notably, the extensive cell enlargement was observed within one week’s treatment and preceded the expression of SA-β-gal.

Senescence can be triggered by different signaling pathways, mainly p16^INK4a^-RB and p53–p21. In the context of GBMs, it is noteworthy that approximately 60% of these tumors harbor deletions or alterations in the *CDKN2A* gene (encoding the p16^INK4a^ protein) (cbioportal.org), which also applies to the U3065MG cell line [6]. BMP4 is a recognized inducer of the CDK inhibitor p21 [29,30], and we indeed observed elevated p21 levels in BMP4-treated cells compared to untreated cells (Figure 1F,G). Moreover, BMP4 treatment induced an increase in lysosomal mass, as evidenced by LAMP2 staining (Figure 1F), and downregulated the nuclear membrane protein lamin B1 (Figure 1H), representing additional features of senescent cells [26,31].

### 2.2. Canonical SMAD Signaling Mediates the BMP4-Induced Senescence-like Phenotype

BMP4 treatment of U3065MG cells activated the downstream canonical SMAD signaling pathway (Supplementary Figure S1A). To investigate its role in senescence induction, we conducted a two-week siRNA-mediated *SMAD4* knockdown (Figure 1I). BMP4-treated *SMAD4* knockdown cells exhibited neither an increase in cell size nor granularity (Supplementary Figure S1B,C), and no downregulation of lamin B1 was observed (Figure 1H). Although SA-β-gal activity was not completely abolished, the minimal levels observed were likely due to incomplete *SMAD4* knockdown (Figure 1J and Supplementary Figure S1D). Together, these findings highlight the essential role of the canonical SMAD pathway in driving the senescence-like phenotype.

### 2.3. BMP4 Induces Transcriptional Programs Related to Mesenchymal Transition and Senescence

We next performed single-sample GSEA (ssGSEA) on bulk RNA sequencing data from U3065MG and *SMAD4* knockdown experiments, using selected gene sets (Supplementary Table S2) related to GBM cell states [2], cell cycling [32], stemness [33], multitherapy resistance [6], cancer MES transition [34], cell size [21], quiescence [35], and senescence induced by various factors [36,37,38,39], as well as MSigDB Hallmarks (Figure 1K, Supplementary Figures S1E and S2). Consistent with the global analysis (Supplementary Table S1), BMP4 treatment downregulated signatures related to cell cycling and neurodevelopmental/PN-associated GBM cell states, including the activity of the stemness transcription factor SOX2, neuronal progenitor cells (NPC1 and NPC2), oligodendrocyte precursor cells (OPC), and astrocyte-like cells (AC) (Figure 1K). In contrast, BMP4 enriched gene sets related to senescence, the MES GBM subtype, MES transition, and multitherapy resistance (Figure 1K). These transcriptional changes were not observed in *SMAD4* knockdown cells (Supplementary Figure S1E). Since cell enlargement is prevalent—and perhaps even causative—in senescent cells [21,40], we analyzed gene signatures connected to cell size, including genes coding for so-called sub- and super-scaling proteins (enriched in small and large cells, respectively) [21]. BMP4 treatment resulted in dramatic shifts in these signatures, consistent with the observed cell enlargement. We also noted that BMP4 upregulated inflammation and injury response-related Hallmark gene signatures, including interferon response, TNFα signaling via NFκB, IL6-JAK-STAT3 signaling, and inflammatory response (Supplementary Figure S2). In conclusion, the activation of the BMP4-SMAD signaling pathway induces MES transition and a senescence-like phenotype in a subpopulation of GBM cells.

### 2.4. High Baseline Levels of Senescence-Associated Genes in MES-like GBM

**Figure 2:**
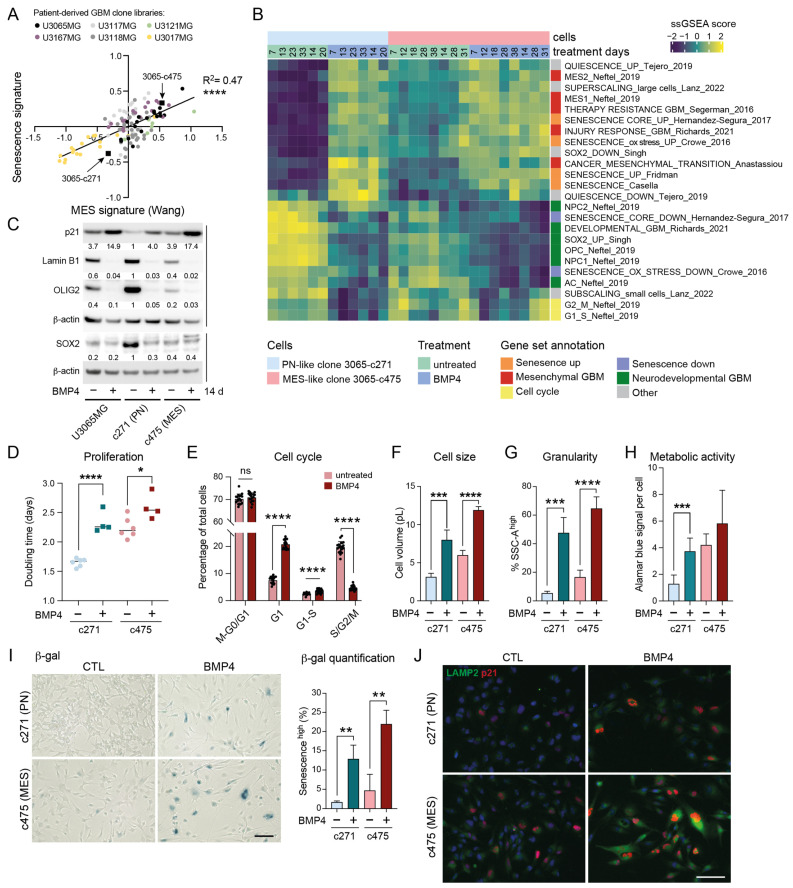
MES-like GBM cells display elevated senescence-associated features compared to PN-like GBM cells. (**A**) A correlation plot of senescence (Fridman, 72 up-regulated genes) and therapy-resistant gene signature scores [6] in 114 GIC clones from five patient tumors (U3167MG, U3117MG/U3118MG, U3121MG, U3065MG, and U3017MG). Signature score was calculated by taking the mean of all individual gene z-scores. ****, *p* ≤ 0.0001. (**B**) A heatmap showing ssGSEA data using the same gene sets as in Figure 1K. (**C**) Western blot analysis of the parental cell line U3065MG and its clonal derivatives 3065-c271 (PN-like) and 3065-c475 (MES-like) cells treated +/−BMP4 for 14 days. The quantification of protein-band intensities is shown, with values normalized to β-actin levels. 3065-c271 was used as reference for comparison. (**D**) The doubling time of 3065-c271 and 3065-c475 cells treated +/−BMP4 for 12–14 days *, *p* = 0.021; ****, *p* ≤ 0.0001. (**E**) A bar graph showing cell-cycle phase distribution of 3065-c475-FUCCI cells 30 h after +/−BMP4 addition. See Supplementary Figure S3F,G. (**F**–**J**) Cells treated for two weeks +/−BMP4. (**F**) Cell volume calculated from cell-diameter measurements. (**G**) Cellular granularity, with a SSC-A high cell population, as in Figure 1. *** = *p* ≤ 0.001; **** = *p* ≤ 0.0001. (**H**) Metabolic activity per cell measured by Alamar blue assay in combination with cell counting. (**I**) SA-β-gal staining—scale bar 100 µm, (left); and the quantification of the CellEventSenescence high cell population from three flow cytometry experiments (right). ** = *p* ≤ 0.01. (**J**) p21 and LAMP2 immunofluorescence staining—scale bar 50 µm.

Given that BMP4 appears to co-regulate genes associated with MES transition and senescence, we next sought to determine whether MES GBMs exhibit a higher baseline expression of senescence-related genes compared to their PN counterparts. We made use of our GBM cell-clone libraries, which include 114 clones derived from five treatment naïve patient tumors, including U3065MG. These clones display a PN-to-MES gradient, which is tightly linked to increasing therapy resistance [6]. A re-analysis of clonal transcriptome data revealed a strong correlation between senescence-related [36] and MES GBM [41] signature genes (Figure 2A). This finding was validated by GSEA on GBM tissue (TCGA) and cell-line data (HGCC, hgcc.se [42]) (Supplementary Figure S3A). Notably, the senescence-regulating gene *CDKN1A* (encoding p21) is part of the MES2 GBM gene signature [2] and is significantly more highly expressed in MES GBM tissue than in other GBM subtypes (TCGA, Gliovis data portal [43]) (Supplementary Figure S3B). Altogether, these data suggest that MES-like tumor cells are inherently closer to a senescent state than PN-like cells.

### 2.5. BMP4 Induces Cell-Cycle Arrest and Senescence-Related Signatures in Both PN-like and MES-like GBM Cells

To explore potential differences in BMP4 responses between PN-like and MES-like GBM cells, we selected two distinct clones from the U3065MG parental cell line: the PN-like clone 3065-c271, and the MES-like clone 3065-c475 (indicated in Figure 2A) [4,6]. These clones expressed high protein levels of the NPC-related marker CD24 and the MES/inflammatory-related marker CD44, respectively (Supplementary Figure S3C) [2]. To examine the potential time-dependent effects of BMP4, both clones were treated with BMP4 for up to 5.5 weeks, with RNA collected at multiple time points (1–5.5 weeks) for bulk RNA sequencing. ssGSEA revealed that BMP4 induced the deregulation of gene sets in both clones similar to those observed in U3065MG (Figure 1K and Figure 2B), including senescence, super-scaling, invasiveness, therapy resistance, and MES GBM gene signatures (Figure 2B). Moreover, BMP4 enriched genes related to increased cellular metabolism, such as the reactive oxygen species (ROS) pathway, as well as injury response and inflammation [5] (Figure 2B and Supplementary Figure S3D). Most of these gene sets had higher baseline levels in the untreated MES-like clone than in the PN-like clone (Figure 2B and Supplementary Figure S3D), underscoring a connection between mesenchymal-, senescence- and stress response-related gene expression.

Concurrently, we observed that BMP4 treatment caused a downregulation of gene sets linked to cell cycling (Figure 2B and Supplementary Figure S3D). This was consistent with increased *CDKN1A* mRNA and p21 protein levels (Figure 2C,J, Supplementary Figure S3E), indicating cell-cycle arrest. Cell counting confirmed BMP4’s anti-proliferative effect, which was more pronounced in the fast-proliferating PN-like 3065-c271 than in the MES-like 3065-c475 (Figure 2D). However, a significant proportion of 3065-c475 cells accumulated in G1 by BMP4, as demonstrated by live cell-cycle phase (FUCCI) monitoring (Figure 2E, Supplementary Figure S3F,G). Moreover, cell-tracing dye administration to the clones on treatment day 8 (+/−BMP4) revealed a subpopulation of non-dividing cells five days later (Supplementary Figure S3H), further supporting BMP4-induced cell-cycle arrest.

### 2.6. Differentiation Effects of BMP4 Are Context Dependent

We have previously shown that BMP4’s anti-proliferative effect is partially explained by SOX2 downregulation [8], which was pronounced in the SOX2-high PN-like clone 3065-c271 (Figure 2C). At the transcriptome level, BMP4 treatment led to a clear reduction in SOX2 activity in both clones (Figure 2B). Furthermore, consistent with the observed downregulation of NPC1- and OPC-related transcripts, BMP4 treatment also reduced OLIG2 protein levels (Figure 2B,C). Although BMP4-mediated suppression of the oligodendrocytic lineage has been linked to astrocytic differentiation [44], we observed a downregulation of the astrocyte-like (AC) gene signature [2] in both clones (Figure 2B), consistent with observations in the parental cell line (Figure 1K). At the protein level, however, the parental cell line and the PN-like clone displayed increased levels of the astrocyte marker GFAP following BMP4 treatment, whereas the MES-like 3065-c475 robustly reduced GFAP levels (Supplementary Figure S3J,K), indicating context-dependent differentiation effects by BMP4.

### 2.7. MES-like GBM Cells Are More Susceptible to BMP4-Induced Senescence than PN-like Cells

BMP4 treatment induced further senescence-associated phenotypes in both clones, including increased cell size—shown by the enrichment of super-scaling genes and larger cell volumes (Figure 2B,E, Supplementary Figure S3I)—and increased cellular granularity (Figure 2G). Senescent cells often show increased metabolic activity compared to both actively dividing cells and quiescent cells, partly due to their high production of SASP factors. Using the Alamar blue assay, which detects metabolic activity through resazurin reduction, we observed increased metabolic activity per cell upon BMP4 treatment (Figure 2H). BMP4-treated cells also exhibited hallmark senescence markers, including increased SA-β-gal activity, p21 and LAMP2 expression, and reduced lamin B1 levels (Figure 2C,I,J). Notably, these markers were most pronounced in MES-like 3065-c475 cells. To further validate this, two additional MES-like, therapy-resistant clones from independent patient tumors (3117-c612 and 3167-c723) [6] were treated with BMP4 for two weeks. Consistent with observations in 3065-c475, BMP4 treatment resulted in significant growth inhibition, elevated SA-β-gal activity and p21 expression, and reduced lamin B1 expression, supporting senescence as a general response to BMP4 in therapy-resistant GBM cells (Supplementary Figure S4).

Overall, these findings demonstrate that untreated p21 high-expressing MES-like cells are intrinsically closer to a senescent state than PN-like cells, experimentally validating transcriptome analyses of GBM tissue and cells. This inherent proximity likely accounts for the heightened susceptibility of MES-like cells to BMP4-induced senescence.

### 2.8. Senescence-Induction by BMP4 Is Dependent on p21

**Figure 3:**
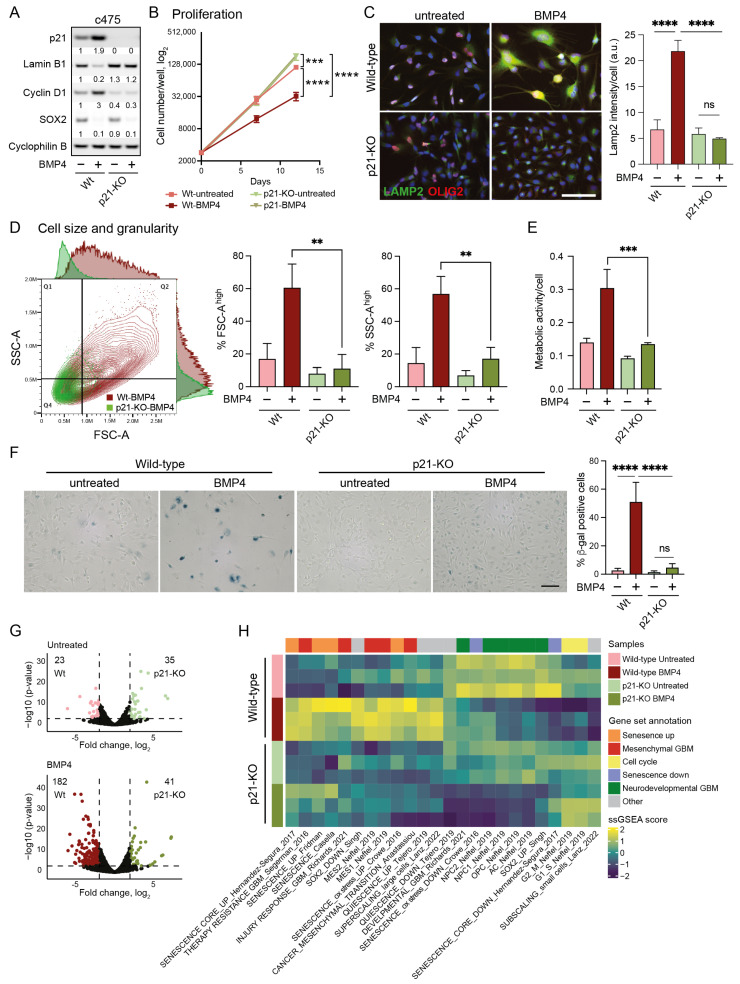
Cell-size enlargement and senescence-like induction by BMP4 is dependent on p21. (**A**–**H**) Wild-type and p21 knockout (p21-KO) MES-like clone 3065-c475 cells, untreated or treated for 12–14 days with BMP4. (**A**) Western blot using antibodies against p21, lamin B1, cyclin D1, SOX2, and cyclophilin B. (**B**) Proliferation; cells counted on days 0, day 7 and day 12. Unpaired *t*-test, *** = *p* ≤ 0.001, ****, *p* ≤ 0.0001. (**C**) Immunocytochemistry using antibodies against LAMP2 and OLIG2 (left). The quantification of LAMP2 signal intensity per cell. Unpaired *t*-test, ****, *p* ≤ 0.0001; ns, not significant (right). (**D**) Flow cytometry analysis of cell size and granularity using FSC-A and SSC-A measurements, respectively (left); and the quantification of FSC-A high and SSC high cell populations (indicated by lines in dot plot), ** = *p* ≤ 0.01 (right). (**E**) Metabolic activity per individual cell. *** = *p* ≤ 0.001. (**F**) SA-β-gal staining photographs— scale bar 100 µm (left); and quantification, ****, *p* ≤ 0.0001; ns, not significant (right). (**G**) Volcano plots of differentially expressed genes (DEGs, 2 ≤ log2 fold difference and *p* ≤ 0.001, thresholds indicated by dotted lines) from wild-type and p21-KO c475 cells, comparing both untreated cells (top, 58 DEGs) and BMP4-treated cells (bottom, 223 DEGs). (**H**) A heatmap of ssGSEA-scores of c475 wild-type and p21-KO cells +/−BMP4. See also Supplementary Figure S5 for data on U3065MG wild-type and p21-KO.

To elucidate the mechanism underlying BMP4-induced senescence, we next examined the specific role of p21. Using the CRISPR-Cas9 system, we generated *CDKN1A* knockout (p21-KO) models in the parental U3065MG cell line and the MES-like clone 3065-c475, chosen for their stronger senescence response compared to the p21 low-expressing PN-like clone 3065-c271. Complete p21-KO cultures were successfully established in both 3065-c475 and U3065MG (Figure 3A and Supplementary Figure S5A). The most pronounced phenotypic effect of p21 deletion was observed in MES-like 3065-c475 cells, which exhibited a marked increase in proliferation compared to wild-type cells (Figure 3B). Both wild-type and p21-KO cells were treated with or without BMP4 for two weeks, followed by phenotypic analyses and bulk RNA sequencing.

In 3065-c475 p21-KO cells, BMP4’s anti-proliferative effect was entirely abolished (Figure 3B), notably despite the downregulation of SOX2 (Figure 3A). Furthermore, the absence of cyclin D1 upregulation in response to BMP4 indicated a failure to induce cell-cycle arrest without p21 (Figure 3A). Most BMP4-induced senescence-related markers were also absent in p21-KO cells, including lamin B1 downregulation (Figure 3A), increased lysosomal mass (Figure 3C), cell enlargement, and enhanced granularity (Figure 3D). Additionally, BMP4-induced increases in metabolic activity (Figure 3E) and SA-β-gal expression (Figure 3F) were essentially abolished in 3065-c475 p21-KO cells. Although the effects were not as pronounced as in 3065-c475 p21-KO cells, similar results were observed in BMP4-treated U3065MG p21-KO cells for most senescence assays (Supplementary Figure S5A–E). These results show that p21 plays a central role in mediating BMP4-induced senescence in GBM cells, particularly in MES-like, therapy-resistant cells.

The transcriptional difference between untreated wild-type and p21-KO cell cultures was modest, with only 58 differentially expressed genes (DEGs) in 3065-c475 (log_2_ fold difference ≥ 2, *p* ≤ 0.001). However, following BMP4 treatment, the number of DEGs between wild-type and p21-KO cells increased substantially to 223 DEGs in 3065-c475 (Figure 3G). Thus, p21 appears to play a minor role in transcriptional regulation under baseline conditions, but has a much larger impact in BMP4-treated cells.

Next, we employed ssGSEA to elucidate biological differences between BMP4-treated p21-KO and wild-type cells, using the previously selected gene signatures (Supplementary Table S2, Figure 2B). In p21-KO cells, BMP4-induced enrichment of senescence- and MES-GBM-related signatures was substantially reduced, with no induction of the super-scaling gene set (Figure 3H, Supplementary Figure S5F). Moreover, cell-cycle- and sub-scaling-related signatures remained unaffected by BMP4 in p21-KO cells. Interestingly, BMP4 reduced neurodevelopmental GBM gene signatures regardless of p21 status, corroborating the observed downregulation of SOX2 and OLIG2 (Figure 3A,C, Supplementary Figure S5A). These findings demonstrate that BMP4-driven senescence, MES transition, and cell enlargement are mediated through p21, while the suppression of the neurodevelopmental GBM axis occurs independently of p21 and is likely tied to SOX2 downregulation.

### 2.9. Computational Prediction Identifies p21-High Cells as Sensitive to Navitoclax

**Figure 4:**
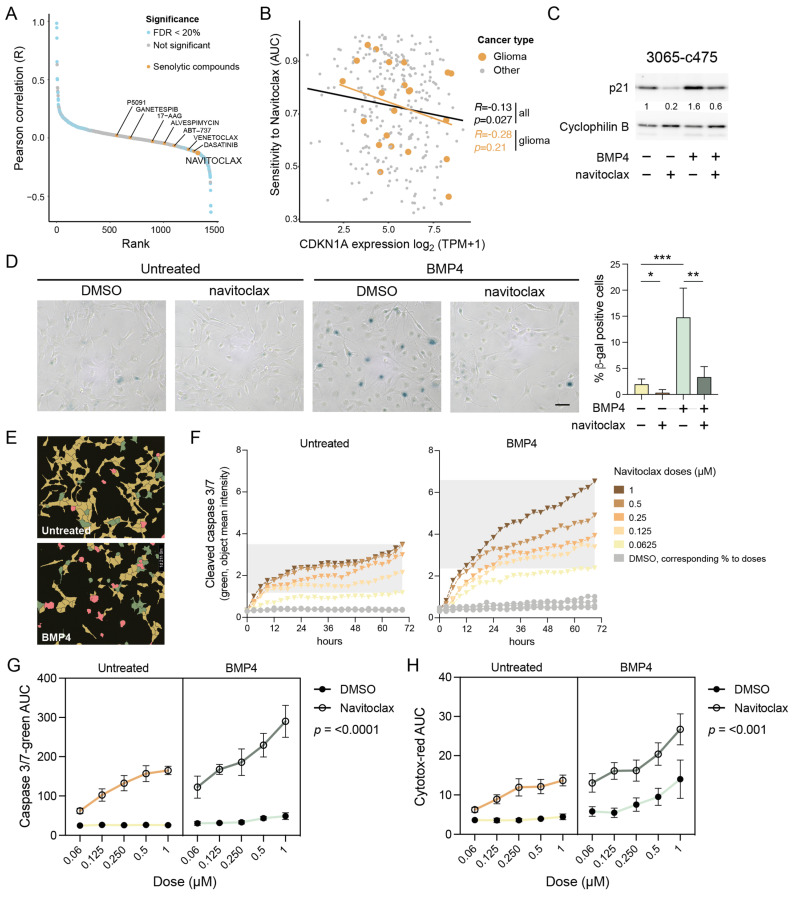
Senolytic treatment eradicates the senescent-like cells via apoptosis. (**A**) Cancer Dependency Map (DepMap) analysis of AUC levels of drugs and compounds and *CDKN1A* gene expression. Senolytic drugs are indicated in yellow. (**B**) The correlation of *CDKN1A* expression and navitoclax sensitivity in cancer cell lines. Gliomas are indicated in yellow. (**C**,**D**) MES-like 3065-c475 cells treated +/−BMP4 for 13 days with navitoclax (0.5 and 0.3 µM, respectively) addition on day 11, analyzed by (**C**) Western blot analysis using antibodies against p21 and cyclophilin B; and (**D**) SA-β-gal staining, scale bar 100 µm (left), and quantification of β-gal-positive cells, normalized values. Unpaired *t*-test, *, *p* = 0.044; **, *p* = 0.001; ***, *p* = 0.0009 (right). (**E**–**H**) Navitoclax treatment (five doses, 0.063–1 µM range) in untreated or BMP4-treated 3065-c475 cells. Navitoclax was added on experimental day 11 and cells were monitored during 72 h. DMSO-treated cells were used as control and cells were analyzed for activity of cleaved caspase 3/7 and cytotoxicity. (**E**) Example photographs of cells (0.125 µM navitoclax for 45 h) showing masks for individual cell analysis. Red, double-positive cells (cleaved caspase 3/7 + cytotoxicity); green, cleaved caspase 3/7 only; yellow, negative cells. (**F**) Cleaved caspase 3/7 apoptosis quantification (object mean intensity) of cells during the 72 h treatment period. (**G**,**H**) AUC values for cleaved caspase 3/7 (**G**) and cytotoxicity (**H**) assays across all +/− navitoclax doses over 72 h. *p*-values denote three-way ANOVA analysis of interaction between +/− navitoclax dose and untreated/BMP4-treated.

To identify potential therapeutic vulnerabilities in p21 high-expressing cells, we next performed a systematic correlation analysis using 1442 cancer cell lines from the Dependency Map dataset [https://depmap.org/portal/ (accessed on 20 April 2024)], integrating RNA expression with drug sensitivity profiles (area under the curve (AUC) values). This unbiased analysis revealed a significant anticorrelation between baseline *CDKN1A*/p21 expression and response to several senolytic agents, suggesting that high p21 levels confer sensitivity to these treatments (Figure 4A). Among these, navitoclax (ABT-263)—an inhibitor of anti-apoptotic Bcl-2/-XL/-w proteins—showed the strongest effect size (R = −0.13; *p* = 0.027) (Figure 4A,B). A similar trend was observed when the analysis was restricted to glioma cell lines only (Figure 4B).

### 2.10. BMP4-Induced Senescent GBM Cells Are Vulnerable to Navitoclax Treatment

To test whether BMP4-treated cells with elevated p21 are more sensitive to navitoclax, MES-like 3065-c475 cells pre-treated with or without BMP4 (11–12 days) were exposed to navitoclax (0.25–0.5 µM) for 48 h. Western blot analysis showed that the combination of BMP4 and navitoclax reduced p21 levels to those of untreated controls (Figure 4C), indicating the selective elimination of BMP4-induced senescent, p21 high-expressing cells. Transcriptomic analysis confirmed reduced expression of senescence-related genes in BMP4 + navitoclax-treated cells compared to BMP4 alone (Supplementary Figure S6). Additionally, the upregulation of cell-cycle-related genes indicated survival of a proliferative, non-senescent subpopulation of cells. Consistent with these findings, SA-β-gal staining revealed a near-complete eradication of the SA-β-gal-positive cell population by navitoclax (Figure 4D).

### 2.11. Navitoclax Triggers Apoptotic Cell Death in BMP4-Induced Senescent GBM Cells

Since navitoclax targets the anti-apoptotic machinery active in senescent cells, we next assessed its apoptotic potential in BMP4-treated cells using a cleaved caspase 3/7 assay. Untreated or BMP4-treated 3065-c475 cells were exposed to navitoclax or DMSO control in five doses (ranging from 0.063 to 1 µM) on treatment day 11. Over 72 h, cells were monitored every three hours for cleaved caspase 3/7 activity and cytotoxicity (plasma membrane integrity), followed by cell-by-cell fluorescence intensity analysis (Figure 4E). BMP4-treated cells exhibited increased susceptibility to navitoclax, as indicated by a sustained rise in apoptosis-related fluorescence intensity over time (Figure 4F). AUC values for cleaved caspase 3/7 and cytotoxicity assays across all doses over 72 h (as shown in Figure 4F) were calculated and analyzed. Three-way ANOVA analyses confirmed a significant difference in navitoclax response—both in terms of apoptosis and cytotoxicity—between untreated and BMP4-treated cells (Figure 4G,H). Notably, BMP4-treated cells also demonstrated increased cytotoxicity to high doses of DMSO, indicating a general decline in plasma membrane integrity. In summary, these findings show that the BMP4-induced senescent subpopulation of GBM cells can be selectively targeted by senolytic treatment.

## 3. Discussion

The development of GBM is driven by dysregulated signaling pathways, with key oncogenic mechanisms including an aberrant activation of PI3K/Akt and Ras/MEK, and the inactivation of *TP53* and p16 (*CDKN2A*)/RB tumor suppressors [45]. While these pathways are well studied, the characterization of inhibitory receptor signaling pathways in GBM remains limited. Among these, BMP signaling has gained attention due to its potential clinical applications (reviewed in [46]), particularly following a recent phase I clinical trial using recombinant human BMP4 treatment in recurrent GBM [12]. This trial demonstrated partial or complete responses in some patients, suggesting that BMP4’s effects are context dependent and could explain the variability in clinical outcome. Our study advances the understanding of BMP4-mediated effects in GBM cells by demonstrating its ability to induce senescence, particularly in therapy-resistant MES-like GBM cells. Importantly, we confirmed this senescence-inducing effect in two additional therapy-resistant clones derived from independent tumors.

### 3.1. Mechanistic Insights into BMP4 Signaling—p21 and Cell Size

In the present study, we demonstrate that canonical BMP4 signaling induces cellular senescence primarily through the activation of p21. Notably, MES-like GBM cells showed greater sensitivity to BMP4-induced senescence compared to PN-like GBM cells, likely due to their elevated baseline levels of p21. The deletion of p21 nearly abolished hallmark features of BMP4-induced senescence, including cell enlargement and SA-β-gal positivity, confirming its essential role. Elevated p21 levels alone are sufficient to induce senescence in various cell lines, including GBM [47,48]. Interestingly, this senescence effect by p21 can be mitigated when cell enlargement is restricted [47], aligning with the smaller size of p21 knockout cells in this study. Abnormal cell enlargement can itself trigger senescence [21,40], as proper cell function relies on maintaining an optimal cell size (reviewed in [49]). Interestingly, pleomorphism—significant cell-size variation—is frequently observed in malignant tumors like GBM, but the mechanisms driving this variation and its impact on tumor progression remain poorly understood. Our data suggest that p21 is a critical regulator of cell size in GBM, linking cell enlargement to heightened senescence susceptibility. Moreover, we found that high *CDKN1A*/p21 levels correlate with increased sensitivity to the senolytic drug navitoclax across cancer cell lines. Navitoclax treatment selectively eliminated SA-β-gal-positive cells through apoptosis, resulting in the restoration of normal p21 levels in surviving cells. Taken together, these findings underscore p21 as a key regulator of BMP4-induced senescence, influencing both cell-size regulation and therapeutic response in GBM.

### 3.2. BMP4 Signaling Divergence—p21 and SOX2

While p21 is necessary for BMP4-induced senescence, our knockout experiments reveal that p21 status does not influence BMP4’s ability to downregulate SOX2. Despite SOX2 downregulation in BMP4-treated p21-KO cells, we observed that cell proliferation remains unaffected. This suggests that BMP4 signaling diverges into distinct pathways, with outcomes depending on cellular context: p21 upregulation drives senescence in some cells, while SOX2 downregulation promotes differentiation in others. Notably, the SOX2 high-expressing PN-like clone upregulated the astrocytic differentiation marker GFAP in response to BMP4, whereas the MES-like clone downregulated GFAP. Interestingly, reduced GFAP expression has also been linked to replicative and oxidative stress-induced senescence in astrocytes [38].

**Figure 5:**
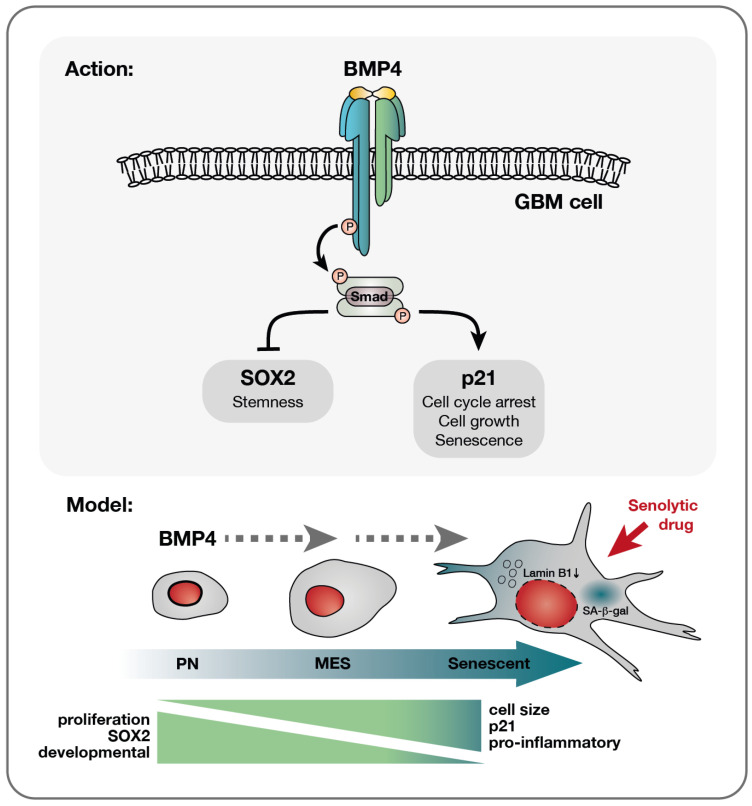
A model of BMP4’s effect on diverse glioblastoma cells—a multifaceted response. Upon the receptor binding of BMP4, the activation of the canonical SMAD complex initiates a bifurcation of the pathway into a SOX2-inhibitory branch and a p21-activating pathway. Consequently, proliferation is inhibited while cellular growth is promoted. This cell-cycle arrest yields different outcomes depending on the cellular state: smaller PN-like GBM cells, characterized by lower basal levels of p21 yet higher SOX2 expression, may preferentially opt for differentiation, as evidenced by astrocytic GFAP upregulation (Supplementary Figure S3J,K). Conversely, larger p21 high-expressing MES-like GBM cells display a higher propensity to undergo senescence in response to BMP4. This senescence-promoting program is characterized by the upregulation of genes, such as pro-inflammatory genes, which are already expressed at elevated levels in these cells compared to their PN counterparts. Importantly, the documented multitherapy-resistant nature of MES-like GBM cells [6] makes their propensity to enter senescence a significant therapeutic opportunity, enabling their targeted elimination through senolytic drug treatment.

Sachdeva et al. previously reported BMP4-induced quiescence in GBM cells [14]. While we observed the enrichment of quiescence-related genes, our data also show the upregulation of genes typically suppressed in quiescent states [35]. Moreover, BMP4 consistently induced senescence-associated features—including SA-β-gal activity, cell enlargement, lamin B1 downregulation, increased metabolic activity, lysosomal mass and granularity—arguing against quiescence in our setting. These findings highlight the heterogenous and context-dependent nature of BMP4 signaling, supporting the hypothesis that BMP4 may drive either differentiation or senescence depending on the basal levels of SOX2 and p21 (Figure 5).

### 3.3. Clinical Implications of Senescence in GBM

The link between therapy resistance and mesenchymal transition in various cancers, including recurrent GBM [50], underscores the need to address intratumoral heterogeneity and cellular plasticity when developing effective therapies. Our prior isolation of multitherapy-resistant and -sensitive clones from the early GBM primary cell cultures [6] enabled us to investigate BMP4’s context-dependent effects. Notably, the therapy-resistant MES-like clones demonstrated greater vulnerability to BMP4-induced senescence than the therapy-sensitive PN-like clone, likely due to higher baseline levels of p21, lower lamin B1 expression, and larger cell size—all characteristics associated with senescence.

The intratumoral PN-to-MES gradient of GBM cells also aligns with an increase in astrocyte reactivity characteristics—a hallmark of the CNS injury response—in the cancer cells [4]. Interestingly, this reactive state is characterized by both cell enlargement and induction of inflammatory factors, many of which overlap with SASP factors [51]. Our pathway analyses of GBM clonal cultures, cell lines, and tissue reveal a molecular link between the MES-like/injury-response/therapy-resistant phenotype and senescence. These findings suggest that GBM cells exist on a spectrum of senescence susceptibility, with the propensity to enter senescence correlating with their position along the PN–MES gradient (Figure 5).

Emerging research underscores the dual role of senescent cells in tumor dynamics (reviewed in [52]). While senescence can impede the progression of premalignant lesions [20], its presence in GBM has been linked to poor prognosis and shorter patient survival [53]. Moreover, radiation-induced senescent cells in the tumor microenvironment can promote tumor growth in GBM mouse models through the release of SASP factors, and the removal of senescent cells has been shown to improve survival in a MES-GBM mouse model [53,54]. Given the tumor-promoting effects of senescent cells, their potential induction by BMP4 should be carefully considered when evaluating BMP4 as a differentiation therapy in a clinical setting.

### 3.4. Future Directions and Conclusions

Our findings highlight BMP4’s ability to induce senescence in MES-like GBM cells with elevated p21 levels and demonstrate that senolytic treatment efficiently removes these senescent cells through apoptosis. Given the therapy-resistant nature of MES-like GBM cells and their propensity to enter senescence, a promising therapeutic strategy could involve a “one-two punch” approach [55]: inducing senescence with a senescence-inducing agent, followed by the targeted elimination of senescent cells using a senolytic drug.

Further investigations into the role of cellular senescence, its interplay with mesenchymal transition, and the impact of senolytic drugs in GBM are warranted, both in treatment-naïve tumors and in recurrences. Importantly, we believe these findings may have broader implications for understanding and treating other cancers beyond GBM.

## 4. Materials and Methods

### 4.1. Cell Culture and Treatments

Cells used in this study were the human GBM cell line U3065MG, obtained from the GBM cell-line biobank HGCC (hgcc.se, [42]), and clonal cultures from early passage U3065MG (3065-c271 and 3065-c475), U3117MG (3117-c612), and U3167MG (3167-723) [6]. The cell line and the clones were tested for authenticity by STR genotyping (AmpF/STR Identifiler PCR Amplification kit, Applied Biosystems, Foster City, CA, USA) and for Mycoplasma contamination (MycoAlert Mycoplasma Detection kit, Lonza, Basel, Switzerland). Cells were grown adherently on laminin (Sigma-Aldrich, Saint Louis, MO, USA)-coated Primaria cell-culture dishes (BD Biosciences, Franklin Lakes, NJ, USA) in serum-free neural stem-cell media (Neurobasal and DMEM/F12 media (1:1) supplemented with N2, B27 w/o retinoic acid (Thermo Fisher Scientific, Waltham, MA, USA), EGF (10 ng/mL) and bFGF (10 ng/mL) (Peprotech, Cranbury, NJ, USA)). For cell detachment, TrypLE Select or TrypLE Express (Thermo Fisher Scientific) was used. Cell counting and cell-diameter measurements were carried out using Trypan blue staining, followed by analysis in an automated cell counter (Countess III, Invitrogen, Waltham, MA, USA). For longterm BMP4 experiments, both sparse seeding (1500–3000 cells/cm^2^) and standard density seeding (1:3 passage, in general 20,000–40,000 cells/cm^2^ depending on cell size) were used. Recombinant human BMP4 (Thermo Fisher Scientific) at 10 ng/mL was added every third day in the presence of EGF and bFGF in the media, in accordance with the treatment scheme used in our previous work [8]. During media change, every three to four days, at least 50% of conditioned media was retained. The senolytic drug navitoclax (ABT-263, Selleck Chemicals, Houston, TX, USA) was used in 0.063–1 µM concentrations for dose–response experiment, and 0.25–0.5 µM was used for Western blot and SA-β-gal activity experiments.

### 4.2. Proliferation, Metabolic Activity, and Cell Cycle Analyses

For proliferation and metabolic activity experiments, cells were seeded sparsely (1500–3000 cells/cm^2^) in 24-well dishes. Each time point and treatment condition included 2–6 replicate wells. BMP4 was added the following day. After two weeks, before cell counting, cellular metabolic activity was assessed using the Alamar blue assay (based on resazurin reduction), following the manufacturer’s instructions. Fluorescence was measured using a Clariostar Plus plate reader (BMG Labtech, Ortenberg, Germany). Samples were counted on indicated days (7–14 days in parallel wells), using a Countess III automated cell counter (Thermo Fisher Scientific). The calculation of cell-doubling time was performed as follows: The growth rate (*r*) was calculated from day 10 or day 12 cell counts, using the formula: $r=\frac{ln(N(t)/N_{0})}{t}$, where *N*(*t*) is the number of cells at time *t*, $N_{0}$ is the number of cells initially seeded, and *t* is the time in culture (in days). From this, the growth rate was converted to doubling time using the formula: $doubling\;time=\frac{ln(2)}{r}$.

For live cell-cycle analysis, 3065-c475 cells were transduced with fluorescent ubiquitination-based cell-cycle indicator (FUCCI; dual Cdt1-TagGFP2 (green) and geminin-TagRFP (orange)) lentivirus (Sartorius AG, Göttingen, Germany) at 3 MOI, followed by puromycin selection (0.5 μg/mL) and cell expansion. 3065-c475-FUCCI cells were seeded in a laminin-coated 24-well tissue culture plate. The day after, fresh media (+/−BMP4) were added and cells were monitored continuously, every 30 min for 72 h, in an Incucyte SX5 Live-Cell Analysis System (Sartorius AG). Cell-by-cell analysis (Incucyte, Sartorius AG) was used for cell segmentation and gate classification of fluorescence intensities.

For measurement of non-dividing (dye-retaining) cells, CellTrace—Violet Cell proliferation Kit (Invitrogen) was used. Cells were incubated with 2 µM CellTrace for 20 min, followed by washing and flow cytometry measurement (405 nm), according to the manufacturer’s protocol. To measure the percentage of dye-retaining cells, a second flow cytometry reading was performed five days later.

### 4.3. Beta-Galactosidase Staining

The detection of SA-β-gal activity on adherent cells was carried out using the Senescence β-Galactosidase Staining Kit (#9860, Cell Signaling Technology, Danvers, MA, USA), according to manufacturer’s instructions. Briefly, cells cultured in 35 mm tissue-culture plates were fixed and stained with an X-gal-containing staining solution (pH 6.0), incubated overnight at 37 °C in a CO_2_-free incubator, then washed with PBS and imaged using a light microscope. For the flow cytometry detection of β-gal, the CellEvent Senescence Green Flow Cytometry Assay Kit (C10840, Invitrogen) was used according to the recommended protocol. Briefly, cells were detached and fixed in 2% paraformaldehyde for 10 min, washed in 1%BSA/PBS and incubated in CellEvent Senescence Working buffer (Invitrogen) containing a 1:1000 probe dilution for 2 h at 37 °C (CO_2_-free).

### 4.4. Flow Cytometry

For live cell-size analysis, cells were detached, pelleted, and resuspended in FACS buffer (0.5% BSA/2 mM EDTA in PBS) and the forward-scatter area (FSC-A) was measured. For live cell staining, fluorophore-conjugated antibodies were added and incubated for 20 min, 4 °C, prior to washing in FACS buffer and flow analysis. The antibodies were CD24-BV421 (BD Pharmingen, Franklin Lakes, NJ, USA, #562789, 2 µL/100 µL) and CD44-FITC (BD Pharmingen, #555478, 5 µL/100 µL). Isotype control antibodies for each fluorophore were from the same companies as the primary antibodies. For β-gal co-staining with p21 Waf1/Cip1, CellEvent Senescence-stained cells were blocked and permeabilized (5% FBS/0.5% saponin or in 5% BSA/0.25% Triton) for 15–30 min, followed by p21 Waf1/Cip1 antibody (dilution 1:500, #2947, Cell Signaling) staining in 1%BSA/PBS, 30 min in room temperature. After FACS buffer washing, cells were incubated with secondary antibody Alexa-fluor-goat-anti-rabbit-350 (Invitrogen), 1:1000 in 1%BSA/PBS-T (0.1%Tween in PBS), at 4 °C for 30 min, followed by washing twice in FACS buffer. Flow cytometry was performed on a BD LSR Fortessa instrument (BD Biosciences) or on a Cytoflex LX instrument using the CytExpert software, version 2.6 (Beckman Coulter, Brea, BA, USA). Flow cytometry data were analyzed using the FlowJo v10 software (BD Biosciences).

### 4.5. Immunofluorescence Staining

Cells were fixed in 4% methanol-free formaldehyde solution (16% stock diluted in PBS, Thermo Scientific) for 15 min, washed in PBS 3 times, permeabilized in 0.3% Triton/PBS for 10 min and incubated with 5% NGS/PBS blocking solution for 1 h. Primary antibodies were diluted in 1%BSA/PBS-T, incubation at 4 °C, overnight. After washing in PBS-T, cells were incubated with secondary antibodies (Alexa-fluor-donkey-anti-mouse-488 or -goat-anti-rabbit-555, Invitrogen), 1:1000 dilution in 1%BSA/PBS-T for 1 h in room temperature, followed by PBS-T washing. Antibodies used were p21 Waf1/Cip1 (rabbit, #2947, Cell Signaling), LAMP2 (mouse, sc-18822, Santa Cruz Biotechnology, Dallas, TX, USA), OLIG2 (rabbit, AB9610, Millipore, Burlington, MA, USA), and GFAP (mouse, MAB3402, Millipore). Photographs were taken using a Leica DMi8 microscope (Wetzlar, Germany).

### 4.6. Western Blot Analysis

Cells were scraped and lysed in 1 X RIPA buffer (Millipore) containing protease and phosphatase inhibitors (cOmplete protease inhibitor and PhosStop, Roche, Basel, Switzerland), incubated on ice for 30 min, spun down at 4 °C for 12 min, followed by protein concentration measurement of the supernatant using the Pierce (BCA) Protein Assay Kit (Pierce, Rockford, IL, USA). For electrophoresis separation, 4–12% Bis-Tris polyacrylamide gradient gels (NuPAGE, Thermo Fisher Scientific) were used under reducing conditions and proteins were transferred to nitrocellulose membranes using the Power Blotter System (Thermo Fisher Scientific). Membranes were blocked in 5% BSA for 1 h and incubated with primary antibodies overnight. After washing with TBS-T (0.05% Tween), incubation with appropriate horseradish peroxidase-labeled antibody (GE Healthcare, Chicago, IL, USA) was performed, followed by TBS-T washing. For chemiluminescent detection, ECL Select Reagent and Amersham Imager 680 (GE Healthcare/Cytiva, Marlborough, MA, USA) were used. Primary antibodies used were p21 Waf1/Cip1 (rabbit, #2947), phospho-Smad 1/5/9 (rabbit, #13820), cyclophilin B (rabbit, #43603) (Cell Signaling); Smad 1 (AF2039, R&D Systems, Minneapolis, MN, USA); lamin B1 (rabbit, ab16048), Cyclin D1 (rabbit, ab16663) (Abcam, Cambridge, UK); OLIG2 (rabbit, AB9610), SOX2 (rabbit, AB5603), and GFAP (mouse, MAB3402) (Millipore); beta-actin (mouse, A5441, Sigma-Aldrich). The quantification of Western blot band intensity was performed in Adobe Photoshop 2024. The background intensity adjacent to each individual band was subtracted. Each sample’s band intensity was normalized to its housekeeping protein-band intensity.

### 4.7. Transcriptome Analysis

Total RNA was extracted using the RNeasy Plus kit (Qiagen, Hilden, Germany) followed by concentration measurement on a Qubit 2.0 fluorometer (Thermo Fisher Scientific) and cDNA synthesis using iScript cDNA synthesis kit (BioRad, Hercules, CA, USA). Semiquantitative RT-PCR was performed using 5 ng cDNA, SsoAdvanced SYBR (BioRad) and primers against SMAD4 (F: AAAACGGCCATCTTCAGCAC; R: AGGCCAGTAATGTCCGGGA, Sigma) and GAPDH PrimePCR primers (BioRad) on a Bio-Rad CFX384 Real-Time 384-well PCR Detection System (BioRad) (95 °C, 2 min denaturation; 39× [95 °C, 0.5 s; 60 °C, 30 s]; 0.5 °C increments (65–95 °C) for 2 s per step.

For RNA sequencing, library preparation (poly(A) selection, Illumina, San Diego, CA, USA) and standard RNA sequencing (Illumina NovaSeq, 2 × 150 bp sequencing configuration, 20 M pair-end reads per sample) was performed at Azenta/Genewiz Life Sciences, Leipzig, Germany. The bulk RNA sequencing datasets are available in the NCBI GEO database, under accession number GSE273244.

Sequencing data were processed by the nf-core/rnaseq pipeline (v1.4.2, GRCh37 genome build, https://github.com/nf-core/rnaseq (accessed on 8 January 2024)) (ref 32055031) and salmon counts were used for downstream analysis. The edgeR package was used for normalization between samples and differential gene-expression analysis [56] (version 3.30.3 within R, version 4.0.2 (http://www.r-project.org (accessed on 8 January 2024))). Gene Set Enrichment Analysis (GSEA) was performed using the Broad Institute GSEA 4.0.3 software with the following settings: Gene set permutation with 1000 permutations, with the Diff of Classes metric for ranking genes (https://www.gsea-msigdb.org/gsea/index.jsp (accessed on 20 January 2024)) [57]. For the sub- and super-scaling gene sets, genes where protein expression had a negative or positive correlation with cell size (|mean protein slope| > 0.4) and protein expression correlated with RNA expression (0.5 < Mean RNA slope/Mean protein slope < 2) were selected from [21].

From bulk RNA sequencing data, we calculated enrichment scores for selected gene signatures (Supplementary Table S2) using single-sample Gene Set Enrichment Analysis (ssGSEA) projections [58]. Here, each ssGSEA enrichment score represents the degree at which a specified gene set is deregulated in each analyzed sample.

### 4.8. siRNA Experiments

Cells were seeded one day prior to siRNA transfection using Lipofectamine RNAiMAX (Thermo Fisher Scientific). siRNA used were Smad4 Silencer Select Pre-designed siRNA (1 nM, #4390824, ID s8403) and negative control siRNA (#4390843) (Thermo Fisher Scientific). After 2 days, RNA was collected and checked for si*SMAD4* knockdown using qRT-PCR and BMP4 was added to the cultures. siRNA knockdown was also checked by qRT-PCR on day 6. Cells were re-transfected on day 8 and used for analyses on day 14 (BMP4 for 12 days).

### 4.9. CRISPR/Cas9 Knockout

For knockout of the p21 gene *CDKN1A*, the p21 Waf1/Cip1 CRISPR/Cas9 KO plasmids (sc-400013, pool of three plasmids with individual 20 nt guide RNA, and a GFP gene) and the p21-specific homology-directed repair (HDR) plasmid containing the puromycin resistance gene and the RFP gene (sc-400013-HDR) were transfected into cells according to the manufacturer’s recommendations (Santa Cruz Biotechnology, Dallas, TX, USA). Only the control CRISPR/Cas9 plasmid (sc-41822, non-targeting 20 nt scramble guide RNA), and Transfection Reagent (TFR) were used as controls in MES-like clone 3065-c475 and U3065MG, respectively. After 4 days, puromycin (0.5 µg/mL) was added to the cells to select for successful CRISPR/Cas9 double-strand breaks.

### 4.10. DepMap Drug Sensitivity Analysis

Gene expression from bulk RNA sequencing data (log_2_(TPM + 1); 23Q4 public release) and drug sensitivity scores (AUC PRISM Repurposing Secondary Screen) were downloaded for 1442 cancer cell lines from the DepMap Portal’s Public 23Q4 release [http://depmap.org/portal/download/custom (accessed on 20 April 2024)]. To establish associations between p21 (*CDKN1A*) expression and drug response, we calculated Pearson correlations between each cell line’s p21 gene expression value and available PRISM drug response data for each tested compound. We ranked these correlations and, after correcting for multiple hypothesis testing, set a cut-off false-discovery rate of 20%.

### 4.11. Dose–Response Experiments

3065-c475 cells (untreated vs. pre-treated with BMP4 for 7 days) were seeded onto a laminin-coated 384-well plate (poly-D-lysine pre-coated, Corning #3768) at 300 cells/well. Four days later, the media +/−BMP4 was changed. The Echo 550 system (Labcyte, Sunnyvale, CA, USA) was used to add five doses of navitoclax/ABT263 (Selleck Chemicals, Houston, TX, USA) (62.5–1000 nM), together with Incucyte Caspase-3/7 Green (4 µM, Sartorius AG) and Incucyte Cytotox Red Dye (0.2 µM, Sartorius AG), indicative of apoptosis and cytotoxicity, respectively. Cells were monitored continuously, every 3 h for 72 h, in an Incucyte SX5 Live-Cell Analysis System (Sartorius AG). Cell-by-cell analysis (Incucyte, Sartorius) was used to measure cell counts and fluorescent dye intensities.

### 4.12. Statistics and Visualizations

GraphPad Prism 10 and R (version 4.0.2 (http://www.r-project.org (accessed on 8 January 2024))) software were used for graph visualizations and statistics. Unpaired *t*-tests were performed to determine significance between two groups. Correlations were calculated using Pearson correlations. Three-way ANOVA analysis was used to determine navitoclax sensitivity between two groups (untreated and BMP4-treated) at different navitoclax doses. Gene-signature scores were received by calculating z-scores for each individual gene in a signature, followed by mean calculations, using Excel.

## Data Availability

The bulk RNA sequencing datasets generated and analyzed during the current study are available in the NCBI GEO database, under accession number GSE273244.

## Supplementary Materials

The following supporting information can be downloaded at: https://www.mdpi.com/article/10.3390/ijms26093974/s1.
