# Supplementary material for: p21-Dependent Senescence Induction by BMP4 Renders Glioblastoma Cells Vulnerable to Senolytics
Source: Int J Mol Sci. 2025 Apr 23;26(9):3974. doi: 10.3390/ijms26093974 (PMC12071447; doi:10.3390/ijms26093974)
Supplement: Supplementary file 1 [file ijms-26-03974-s001.zip › Supplementary Figures_Table S1_Niklasson et al.pdf]

Supplementary figures

Supplementary Figure S1

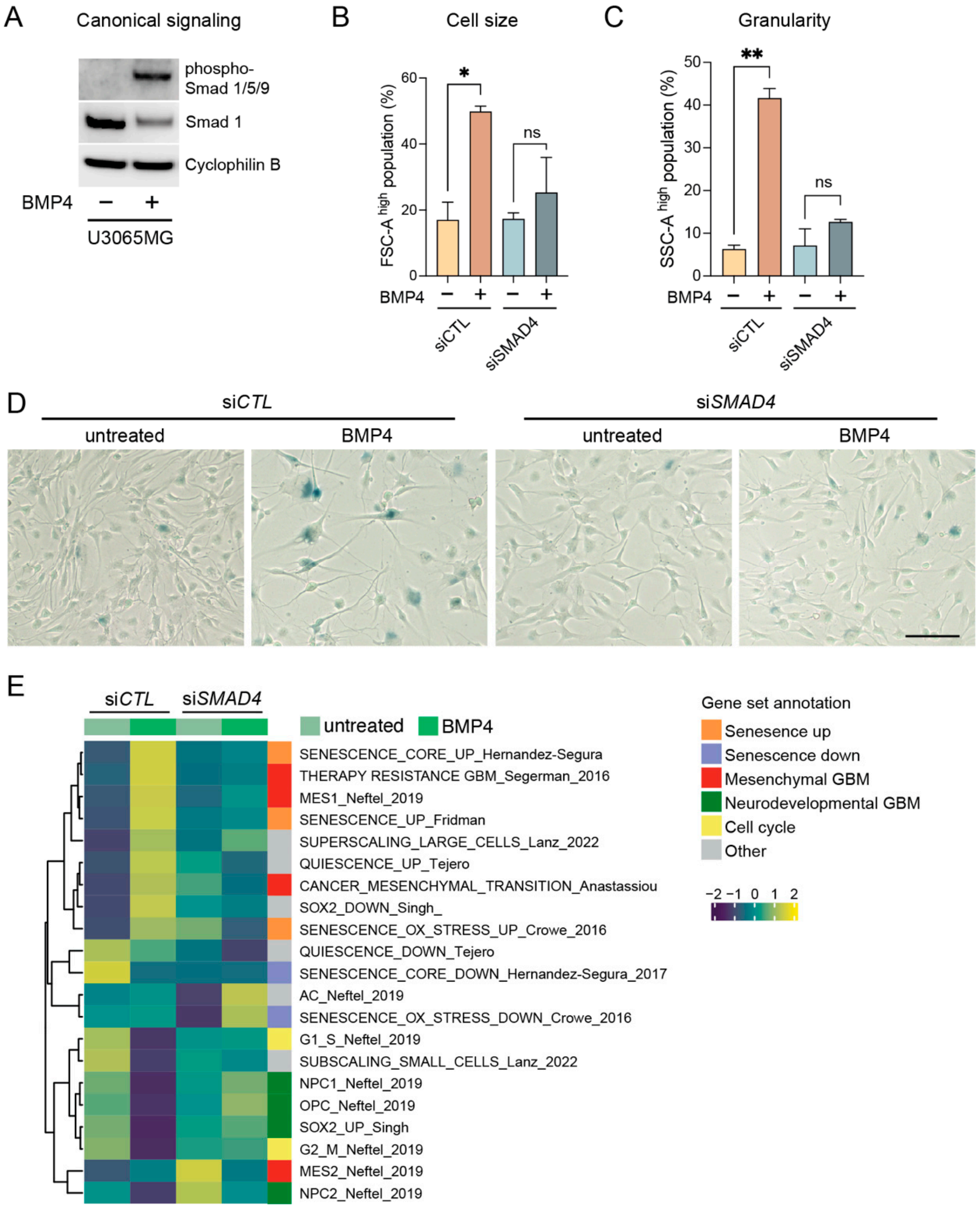

Supplementary Figure S1 *(connected to Figure 1). A. Phospho-SMAD1/5/9 and total SMAD1 western blot analysis of U3065MG cells stimulated with BMP4 (10 ng/mL) for 1 hour. B-E. siRNA-mediated knock-down of SMAD4 in U3065MG cells. Cells were transduced two times with siRNA against*

*SMAD4 or scrambled control (siCTL) (day 0 and day 8), with addition of +/- BMP4 on day 2. BMP4 treatment for 12 days. B-C. Flow cytometry analysis of B) cellular size, gating on the forward-scatter-high cell population (FSC-A high), and C) cellular granularity, gating on the side scatter-high cell population (SSC-A high), as in Figure 1B-C. Two experiments, unpaired t-test, *, p=0.014; **, p=0.0024. D. SA-β-gal staining photographs of U3065MG cells +/-BMP4 (14 days), scale bar 100 µm. E. Single-sample Gene Set Enrichment Analysis (ssGSEA) of transcriptome data from SMAD4 knockdown experiments.*

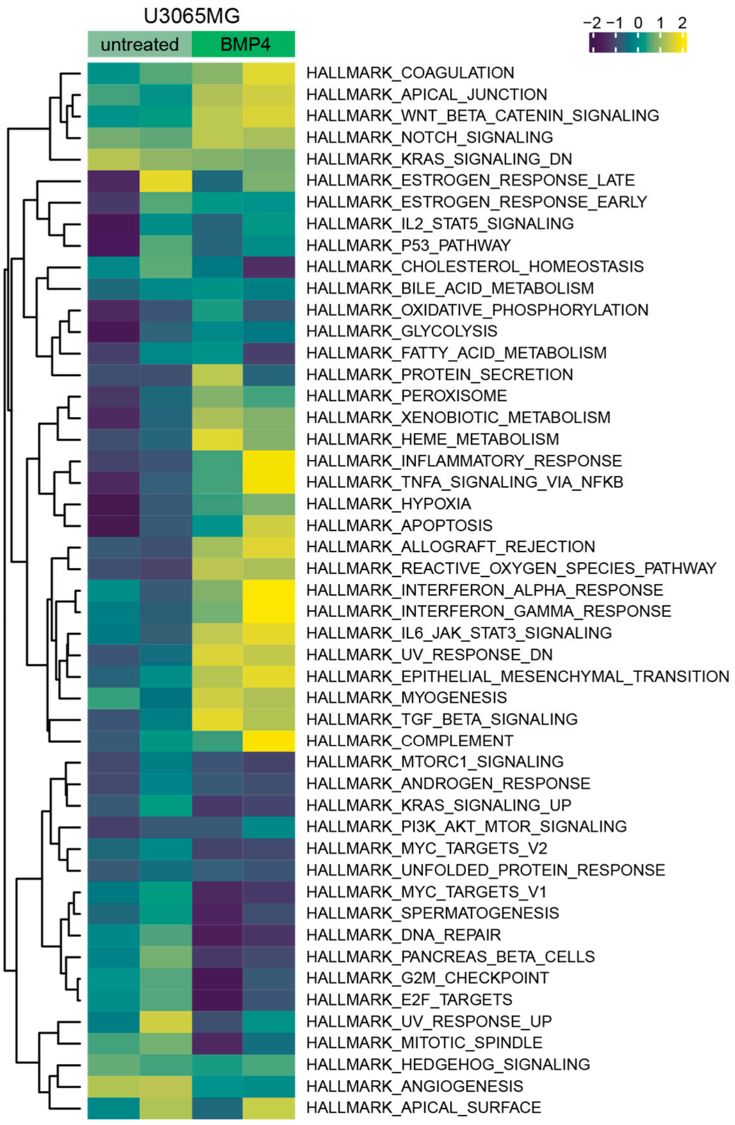

Supplementary Figure S2 *(connected to Figure 1). Heatmap of ssGSEA MSigDB Hallmark analysis on transcriptome data from untreated or BMP4-treated (14 days) U3065MG cells, two experiments.*

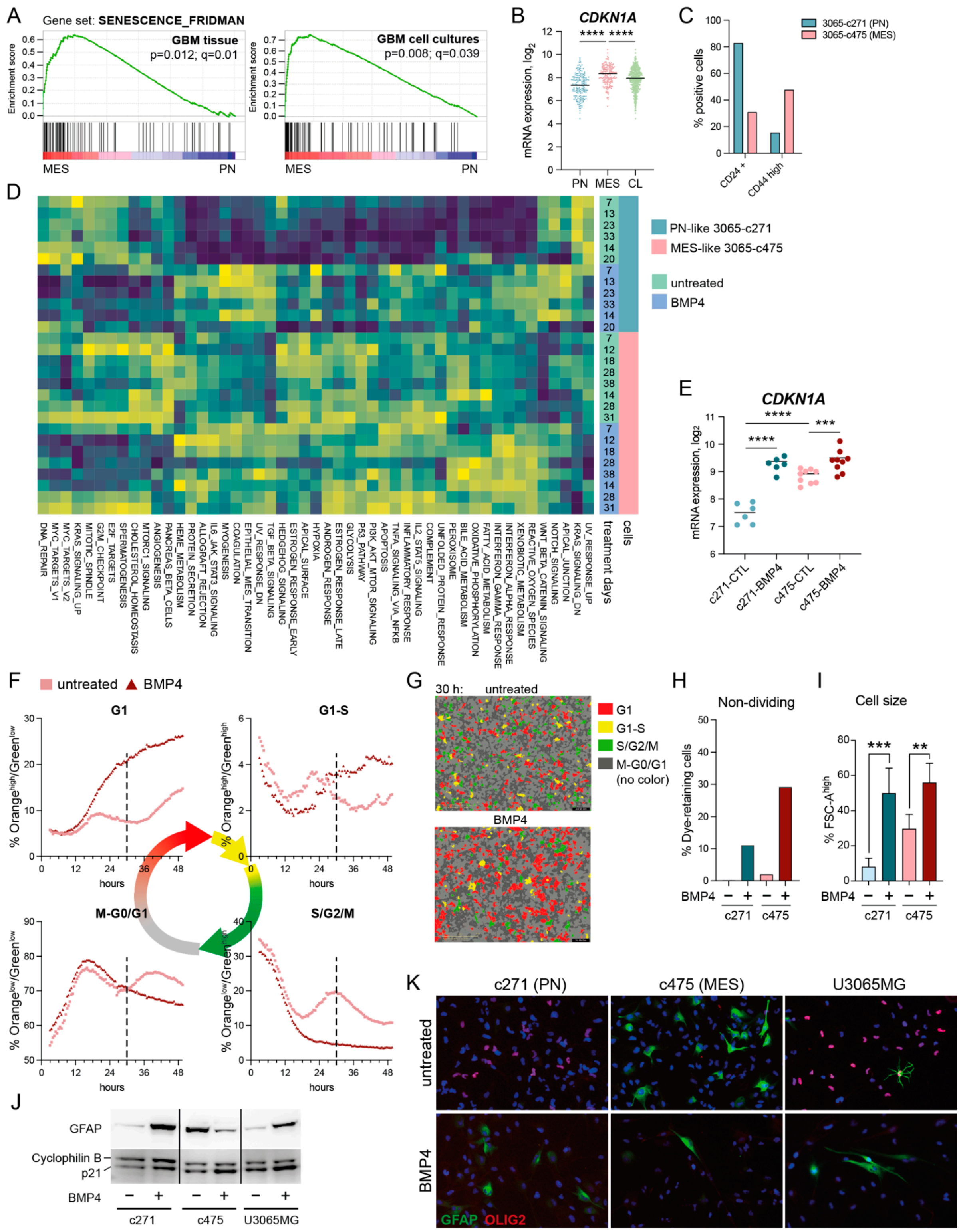

Supplementary Figure S3 *(connected to Figure 2). A. GSEA using the Fridman Senescence Up gene set on mesenchymal (n=158) vs. proneural (n=139) GBM tumors from TCGA database (left); and 19 mesenchymal vs. 14 proneural GBM cell lines from the HGCC repository (right). B. CDKN1A (p21 gene) expression in GBM tissue (TCGA, Gliovis data portal [43], unpaired t-test, ****, p-value < 0.0001). C. Bar graph showing percentage of CD24 and CD44-high expressing cells in PN-like 3065-c271 and MES-like 3065-c475 cells using flow cytometry analysis. D. Single sample GSEA using*

*MSigDB Hallmarks gene sets in untreated and BMP4-treated (7-33 days) PN-like clone 3065-c271 and MES-like clone 3065-c475. E. CDKN1A mRNA expression. F-G. 3065-c475-FUCCI cells +/- BMP4 monitored for 48 hours in an Incucyte SX5 instrument followed by cell-by-cell analysis and cell cycle phase gating. Photographs in G shows the cell cycle phase classification masks at the 30h timepoint (data plotted as a bar graph in main Fig. 2E). H. Flow cytometry measurement of CellTrace Violet dye-retaining (non-dividing/cell cycle arrested) cells of 3065-c271 and 3065-c475 cells treated +/-BMP4 for 14 days with dye-incubation during the last 5 days. I. Quantification of flow cytometry FSC-A high cell population, as in Figure 1. ** = p≤0.01; *** = p≤0.001. J-K. GFAP western blot (J) and OLIG2 and GFAP immunofluorescence staining (K) of U3065MG, 3065-c271 and 3065-c475 treated +/-BMP4 for two weeks.*

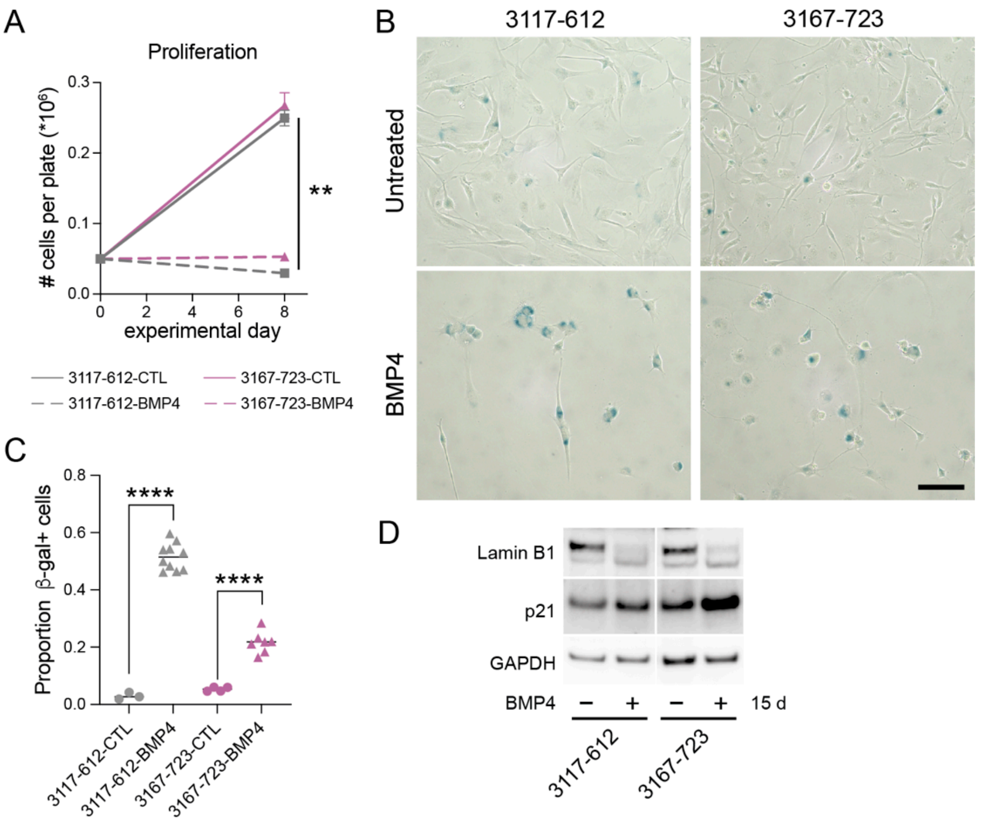

Supplementary Figure S4 *(connected to Figure 2). Therapy-resistant, MES-like clones from two independent patient tumors, 3117-c612 and 3167-c723 [6], were seeded at 50 000 cells per 35 mm tissue culture plate and treated +/- BMP4. After eight days, cells were counted (A) and untreated cultures were passaged. After 15 days, cells were stained and quantified for SA-β-gal expression (B-C; scale, 100 µm), and analyzed for lamin B1 and p21 levels by western blot analysis (D).*

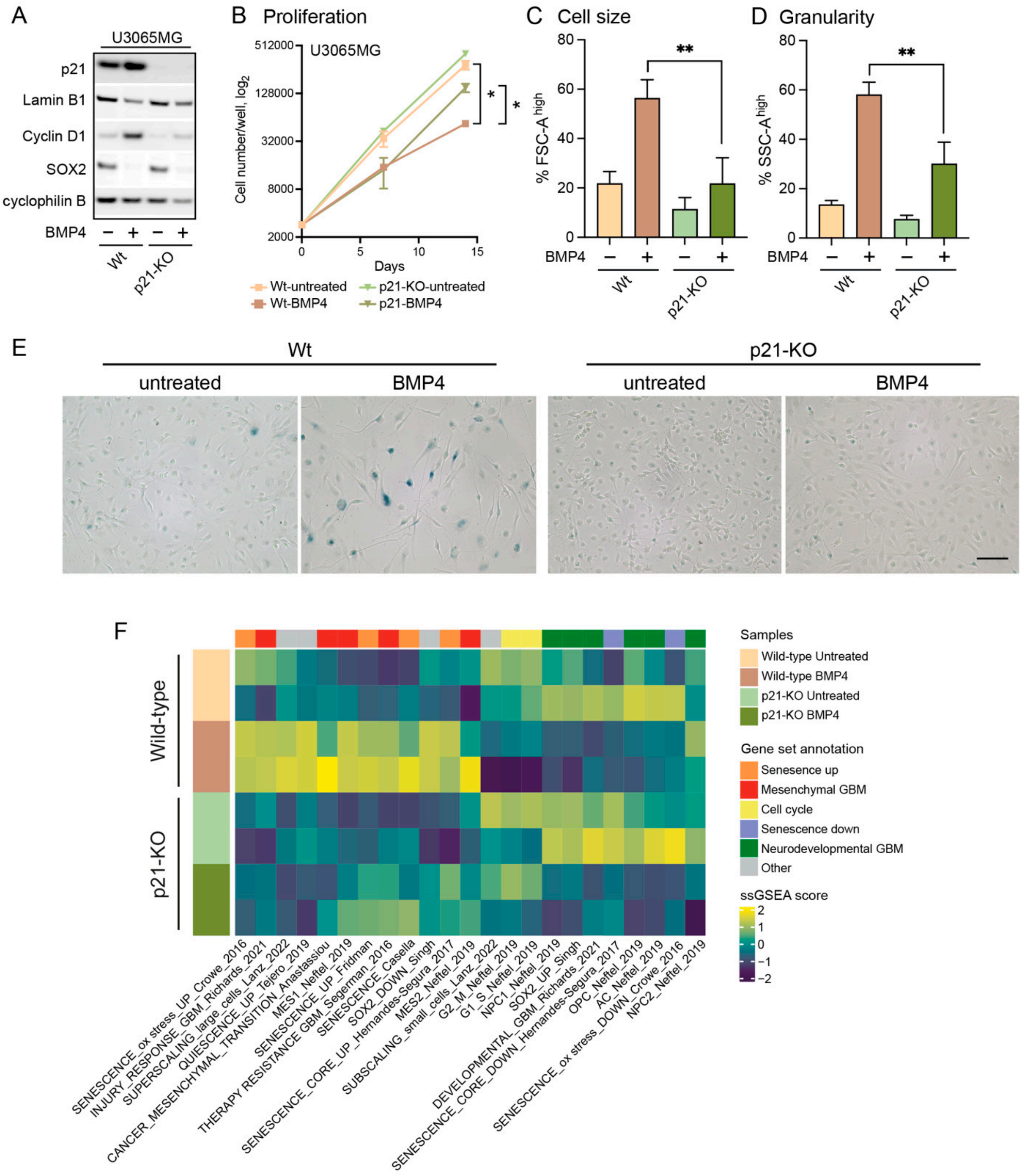

Supplementary Figure S5 *(connected to Figure 3). A. Western blot on wild-type and p21 knockout (p21-KO) U3065MG cells +/- BMP4 using antibodies against p21, lamin B1, cyclin D1, SOX2, and cyclophilin B. B. Proliferation of wild-type and p21-KO cells +/- BMP4. Cell counting on day 0, day 7 and day 14. C. Flow cytometry analysis of cell size (cell growth) using forward scatter (FSC-A) measurements and quantification of the FSC-A high cell population; and cell granularity (D). Side scatter area (SSC-A) high gated population is plotted. E. SA-β-gal staining, scale bar 100 µm. F. Heatmap of ssGSEA-scores of U3065MG wild-type and p21-KO cells +/-BMP4.*

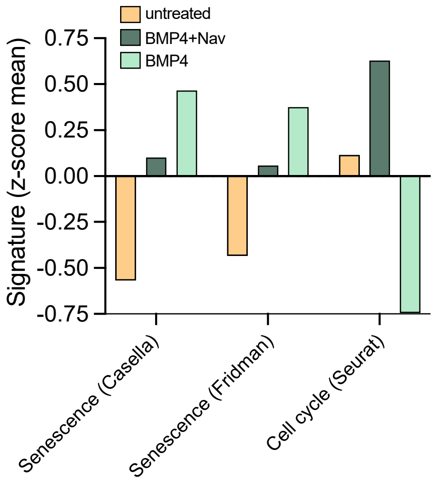

Supplementary Figure S6 *(connected to Figure 4). Bar graph showing senescence [36, 39] and cell cycle [32] gene signature scores (mean z values) of untreated, and BMP4-treated +/- navitoclax 3065-c475 cells.*

Supplementary Table S1. GSEA on U3065MG +/-BMP4 (10 ng/ml), 12-13 days.

producer_class	xtools.gsea.GseaPreranked	
scoring_scheme	weighted	
norm	meandiv	
nperm		1000

GSEA on U3065MG -/+ BMP4 10 ng/ml for 13-14 days.
n = 4 samples/condition
Ranked gene list where ranking metric = log2(FC)*(-log10(p-val))
Gene set: GSEA C2. Chemical and Genetic pertubations (ftp.broadinstitute.org://pub/gsea/gene_sets/c2.cgp.v2023.2.Hs.symbols.gmt)

BMP4 DOWNREGULATED TRANSCRIPTS

	GS follow link to MSigDB	SIZE	ES	NES	NOM p-val	FDR q-val	FWER p-val	RANK AT MAX	LEADING EDGE
1	VERHAAK_GLIOBLASTOMA_PRONEURAL	131	-0.88	-1.66	0.000	0.199	0.181	559	tags=26%, list=5%, signal=27%
2	FISCHER_G2_M_CELL_CYCLE	206	-0.84	-1.65	0.003	0.145	0.252	817	tags=41%, list=7%, signal=44%
3	SOTIRIOU_BREAST_CANCER_GRADE_1_VS_3_UP	142	-0.87	-1.65	0.000	0.107	0.274	884	tags=56%, list=7%, signal=60%
4	ROSTY_CERVICAL_CANCER_PROLIFERATION_CLUSTER	125	-0.89	-1.64	0.003	0.124	0.389	1216	tags=73%, list=10%, signal=80%
5	VANTVEER_BREAST_CANCER_METASTASIS_DN	95	-0.89	-1.62	0.003	0.168	0.562	797	tags=34%, list=7%, signal=36%
6	KOBAYASHI_EGFR_SIGNALING_24HR_DN	235	-0.82	-1.62	0.000	0.149	0.582	926	tags=48%, list=8%, signal=51%
7	WHITEFORD_PEDIATRIC_CANCER_MARKERS	106	-0.87	-1.60	0.003	0.255	0.838	1088	tags=63%, list=9%, signal=69%
8	MORI_IMMATURE_B_LYMPHOCYTE_DN	84	-0.86	-1.59	0.015	0.288	0.900	922	tags=52%, list=8%, signal=56%
9	ODONNELL_TFRC_TARGETS_DN	98	-0.86	-1.57	0.015	0.370	0.961	773	tags=47%, list=6%, signal=50%
10	GAVIN_FOXP3_TARGETS_CLUSTER_P6	75	-0.88	-1.57	0.015	0.352	0.968	739	tags=51%, list=6%, signal=54%
11	BENPORATH_PROLIFERATION	137	-0.83	-1.57	0.000	0.346	0.977	935	tags=37%, list=8%, signal=40%
12	BUFFA_HYPOXIA_METAGENE	45	-0.92	-1.56	0.009	0.383	0.990	595	tags=20%, list=5%, signal=21%
13	BARRIER_COLON_CANCER_RECURRENCE_UP	31	-0.96	-1.55	0.007	0.418	0.994	5	tags=3%, list=0%, signal=3%
14	BIDUS_METASTASIS_UP	192	-0.81	-1.55	0.003	0.409	0.995	1223	tags=29%, list=10%, signal=31%
15	WU_APOPTOSIS_BY_CDKN1A_VIA_TP53	50	-0.89	-1.55	0.012	0.418	0.999	817	tags=46%, list=7%, signal=49%
16	CHESLER_BRAIN_QTL_CIS	61	-0.88	-1.54	0.021	0.469	1.000	449	tags=13%, list=4%, signal=14%
17	LEE_LIVER_CANCER_SURVIVAL_DN	155	-0.81	-1.54	0.009	0.443	1.000	773	tags=15%, list=6%, signal=16%
18	PURBEY_TARGETS_OF_CTBP1_AND_SATB1_UP	60	-0.88	-1.53	0.007	0.484	1.000	268	tags=3%, list=2%, signal=3%
19	ACOSTA_PROLIFERATION_INDEPENDENT_MYC_TARGETS_UP	66	-0.87	-1.53	0.016	0.467	1.000	326	tags=8%, list=3%, signal=8%
20	FERREIRA_EWINGS_SARCOMA_UNSTABLE_VS_STABLE_UP	136	-0.81	-1.53	0.017	0.462	1.000	1002	tags=31%, list=8%, signal=33%

BMP4 UPREGULATED TRANSCRIPTS

	GS follow link to MSigDB	SIZE	ES	NES	NOM p-val	FDR q-val	FWER p-val	RANK AT MAX	LEADING EDGE
1	REN_ALVEOLAR_RHABDOMYOSARCOMA_DN	380	0.83	1.56	0.000	0.136	0.125	1023	tags=21%, list=9%, signal=23%
2	RIZKI_TUMOR_INVASIVENESS_2D_UP	48	0.93	1.48	0.010	1.000	0.995	3	tags=2%, list=0%, signal=2%
3	YAO_TEMPORAL_RESPONSE_TO_PROGESTERONE_CLUSTER_7	60	0.92	1.47	0.005	1.000	0.999	123	tags=5%, list=1%, signal=5%
4	WATANABE_RECTAL_CANCER_RADIOTHERAPY_RESPONSIVE_DN	83	0.89	1.46	0.013	1.000	0.999	305	tags=7%, list=3%, signal=7%
5	SENESE_HDAC1_AND_HDAC2_TARGETS_DN	162	0.84	1.46	0.006	1.000	1.000	436	tags=16%, list=4%, signal=16%
6	GENTILE_UV_RESPONSE_CLUSTER_D5	34	0.94	1.46	0.018	1.000	1.000	119	tags=6%, list=1%, signal=6%
7	WANG_ESOPHAGUS_CANCER_VS_NORMAL_UP	84	0.88	1.45	0.014	1.000	1.000	573	tags=18%, list=5%, signal=19%
8	PICCALUGA_ANGIOIMMUNOBLASTIC_LYMPHOMA_UP	135	0.85	1.45	0.004	1.000	1.000	493	tags=23%, list=4%, signal=24%
9	LEE_AGING_MUSCLE_DN	29	0.94	1.45	0.011	1.000	1.000	42	tags=10%, list=0%, signal=10%
10	IVANOVA_HEMATOPOIESIS_MATURE_CELL	198	0.80	1.45	0.012	1.000	1.000	326	tags=4%, list=3%, signal=4%
11	BOQUEST_STEM_CELL_UP	179	0.81	1.45	0.017	1.000	1.000	521	tags=28%, list=4%, signal=29%
12	FRIDMAN_SENESCENCE_UP	64	0.89	1.45	0.021	1.000	1.000	573	tags=22%, list=5%, signal=23%
13	MEISSNER_BRAIN_HCP_WITH_H3K27ME3	65	0.89	1.44	0.027	1.000	1.000	155	tags=11%, list=1%, signal=11%
14	RODWELL_AGING_KIDNEY_NO_BLOOD_UP	154	0.82	1.44	0.018	1.000	1.000	673	tags=19%, list=6%, signal=20%
15	VERHAAK_GLIOBLASTOMA_MESENCHYMAL	136	0.84	1.43	0.020	1.000	1.000	962	tags=30%, list=8%, signal=32%
16	RAMASWAMY_METASTASIS_UP	58	0.88	1.43	0.025	1.000	1.000	42	tags=3%, list=0%, signal=3%
17	TAKEDA_TARGETS_OF_NUP98_HOXA9_FUSION_6HR_UP	46	0.91	1.43	0.022	1.000	1.000	358	tags=20%, list=3%, signal=20%
18	BUCKANOVICH_T_LYMPHOCYTE_HOMING_ON_TUMOR_UP	15	0.97	1.42	0.013	1.000	1.000	138	tags=20%, list=1%, signal=20%
19	GAURNIER_PSMD4_TARGETS	28	0.94	1.42	0.028	1.000	1.000	496	tags=54%, list=4%, signal=56%
20	HUMMERICH_SKIN_CANCER_PROGRESSION_DN	75	0.87	1.42	0.027	1.000	1.000	436	tags=11%, list=4%, signal=11%
